# Development and Investigation of a Nanoemulgel Formulated from Tunisian *Opuntia ficus-indica* L. Seed Oil for Enhanced Wound Healing Activity

**DOI:** 10.3390/gels10090582

**Published:** 2024-09-09

**Authors:** Badr Bahloul, Enis Ben Bnina, Dorra Dridi, Aya Bouhamed, Luis Castillo Henríquez, Guido Flamini, José Roberto Vega-Baudrit

**Affiliations:** 1 Drug Development Laboratory LR12ES09, Faculty of Pharmacy, University of Monastir, Monastir 5000, Tunisia; drididorraph@gmail.com (D.D.); ayabouhamed@gmail.com (A.B.); 2 LR21AGR03-Production and Protection for a Sustainable Horticulture (2PHD), IRESA-University of Sousse, Regional Research Centre on Horticulture and Organic Agriculture, Sousse 4042, Tunisia; benbninae@gmail.com; 3 CNRS, INSERM, UTCBS, Chemical and Biological Technologies for Health Laboratory, Université Paris Cité, F-75006 Paris, France; luis.castillo-henriquez@etu.u-paris.fr; 4 Dipartimento di Farmacia, Via Bonanno 6, 56126 Pisa, Italy; guido.flamini@unipi.it; 5 National Nanotechnology Laboratory (LANOTEC), National Center for High Technology (CeNAT), San José 1174-1200, Costa Rica; jose.vega.baudrit@una.cr

**Keywords:** pharmaceutical nanotechnology, phytotherapy, nanoemulsion, gas chromatography, *Opuntia ficus-indica* L., advanced gels, topical delivery

## Abstract

The aim of this study is to develop a nanoemulgel encapsulating a Tunisian Prickly Pear (*Opuntia ficus-indica* L.) seed oil (PPSO) to assess, for the first time, the in vivo efficacy of this nanoformulation on wound healing. Phytocompounds of this oil have been reported in the literature as having powerful pharmacological activities. However, it remains poorly exploited due to low bioavailability. A nanoemulsion (NE) was designed by determining the required hydrophilic–lipophilic balance (HLB) and subsequently characterized. The mean droplet size was measured at 56.46 ± 1.12 nm, with a polydispersity index (PDI) of 0.23 ± 0.01 using dynamic light scattering. The zeta potential was −31.4 ± 1.4 mV, and the morphology was confirmed and assessed using transmission electron microscopy (TEM). These characteristics align with the typical properties of nanoemulsions. The gelification process resulted in the formation of a nanoemulgel from the optimum nanoemulsion. The high wound healing efficiency of the nanoemulgel was confirmed compared to that of a medicinally marketed cream. The outcomes of this research contribute valuable insights, for the first time, into the potential therapeutic applications of PPSO and its innovative pharmaceutical formulation for wound healing.

## 1. Introduction

Each year, millions of people worldwide suffer from acute and chronic wounds, making the burden of treatment a major health issue. The increase in acute, surgical, and chronic wounds is expected to cause a significant rise in the total cost of wound care, reaching up to 18.7 billion dollars by 2027. In addition, a growing proportion of the healthcare budget will be devoted to the treatment of chronic wounds [1]. This high cost encourages researchers to find alternatives in order to reduce the expense while obtaining better results. 

Wound healing represents a natural physiological mechanism that initiates wound restoration and recovery of the skin’s integrity. It is one of the body’s most complex and dynamic processes and involves the synchronization of various cell types, growth factors, extracellular matrix components, cytokines, and chemokines. These elements must coordinate through four sequential, overlapping, and interdependent stages, hemostasis, inflammation, proliferation, and remodeling, as well as interactions between numerous cells and cell mediators to ultimately achieve healing [2]. This process is extremely slow and is accompanied by an increased risk of microbial infection, making extracorporeal assistance essential for speeding up its progress [3].

The treatments currently available include enzyme-based ointments, such as DNase and collagenase, as well as polyurethane dressings, hyaluronic acid hydrogels, and synthetic growth factors. However, many of these options are expensive and require prolonged treatment [3].

Nowadays, herbal drugs are gaining popularity, which can be attributed to several factors. Firstly, plant-based extracts are considered natural and have a long history of use in traditional medicine for treating a multitude of conditions. Secondly, plant-derived products are often perceived as safer than synthetic drugs due to their supposedly reduced side effects [1]. Thirdly, these herbal preparations are usually more economical than their synthetic counterparts, especially in low- and middle-income countries. Lastly, the intersection of nanotechnology and natural product chemistry has led to new delivery systems for bioactive compounds extracted from plants, potentially enhancing their efficacy and safety [4].

*Opuntia ficus-indica*, widely known as prickly pear or Nepal cactus, remains a domestic cactus species belonging to the Cactaceae family. The genus *Opuntia* includes over 1500 species [5] distributed in North Africa, the Mediterranean area [6], Europe, America, and the Middle East [7].

The contribution of *Opuntia ficus-indica* to traditional medicine is attributed to its high potential of active compounds, including organic and phenolic acids, fatty acids, phytosterols, saponins, flavonoids, vitamins, betalains, and carotenoids [8]. This wealth exhibits various biological activities such as antiviral [9], antioxidant [10], anti-ulcer [11], antidiabetic [12], analgesic [13], anti-inflammatory [14], hepatoprotective [15] and anticancer effects [16]. In Tunisia, the annual production of edible *Opuntia ficus-indica* fruits is estimated at 1,200,000 tons. The seeds represent sources of natural fiber, constituting 10 to 15% of the fruit’s mass [17]. 

Given its wealth of bioactive compounds, *Opuntia ficus-indica* presents a promising alternative for developing innovative wound healing formulations. For instance, a study demonstrated that a base cream containing 15% lyophilized cladodes of *Opuntia ficus-indica* significantly accelerated wound healing in rats by promoting the proliferation and migration of keratinocytes [18]. More recent research has investigated the anti-inflammatory and wound healing effects of polysaccharide extracts from *Opuntia ficus-indica* cladodes. The study found that topical application of this extract reduced inflammation, increased fibroblast and myofibroblast counts, and enhanced neoangiogenesis, ultimately improving wound healing in cutaneous wounds [19]. Additionally, *Opuntia ficus-indica* extracts have demonstrated antioxidant and antibacterial properties, further contributing to their potential in treating various health conditions, including skin ulcerations [20]. A recent study has also highlighted the effectiveness of PPSO in promoting collagen synthesis and accelerating wound healing [21].

However, this natural therapy based on herbal drugs or conventional forms containing plant extracts has several limitations, such as the lipophilicity of the phytoextract leading to low solubility, easy degradation of the phytomolecules, difficulty in crossing biological barriers, and physicochemical instability [22]. To overcome these drawbacks, new strategies aim to develop innovative treatments by combining traditional healing agents with modern formulation technologies, with the aim of obtaining effective phyto-drug delivery systems [23].

In this field, the use of nanotechnology is a powerful alternative to reduce limitations of conventional phytoformulations. One of the most promising approaches is the use of nanoemulsions, which serve as effective nanocarriers in wound healing [20,24,25].

A nanoemulsion is a type of emulsion in which the droplets are generally between 20 and 200 nanometers (nm) in size. They are composed of oil, water, and an emulsifier (surfactant). Usually, NEs are obtained by high-energy emulsification processes and an optimal NE is formed when the oil and aqueous phases, the hydrophobic–lipophilic balance (HLB), and the percentage of surfactants are carefully adjusted and properly combined [26,27].

High stability is established when the HLB value of a surfactant mixture is close to that required of the oil, which leads to a smaller droplet size with a reduced size distribution [28]. 

These nanoemulsions offer a range of advantages, including improved permeability, controlled drug delivery, enhanced stability, and targeted action. They interact effectively with the skin’s lipid layer, which boosts their effectiveness. A recent study showed that nanoemulsions considerably accelerate the wound healing function by stimulating angiogenesis, decreasing lipid oxidation and inflammation, and improving re-epithelialization [4]. Wounds treated with these nanoemulsions showed increased collagen levels, which contributed to faster healing [29]. Additionally, nanoemulsions were shown to significantly boost the proliferation of fibroblast cells, further reducing the time required for wound closure [27].

Nanoemulgel is an exciting new formulation that merges the advantages of nanoemulsions and hydrogels, making it especially effective for wound healing. This combination not only boosts the delivery of active ingredients but also enhances their solubility and ability to penetrate the skin. Their three-dimensional polymeric networks and porous structure allow for the absorption of aqueous fluids, preventing wound dehydration, and fostering a moist environment conducive to healing [28].

Recent research has demonstrated the effectiveness of nanoemulgel systems that incorporate herbal extracts in wound healing applications. For example, the ethanol extract of *Artocarpus lakoocha* Roxb. (mobe leaves), rich in flavonoids and tannins, has been formulated into nanoemulgel systems, showing significant potential in promoting fibroblast proliferation and speeding up the healing process [30]. Additionally, tea tree oil has been integrated into nanoemulgel formulations and often combined with other agents, like mupirocin, to enhance its antibacterial properties and accelerate wound healing [31]. Similarly, lemongrass oil, known for its antibacterial and antioxidant properties, has been utilized in a topical nanoemulgel preparation, with added ferulic acid to boost its wound healing efficacy [32].

As part of the research on natural treatments for wound healing, our study aimed to evaluate the therapeutic effect of Tunisian *Opuntia ficus-indica* seed oil (PPSO) and its nanoemulgel formulation and to provide valuable insights into wound healing, an area that, to our knowledge, has not been previously explored. Therefore, our research is the first of its kind to explore PPSO under an innovative formulation. 

We embarked on the formulation of the nanoemulsion by employing a carefully selected mixture of surfactants. This was followed by extensive characterization, including stability assessment, droplet size and zeta potential measurements, and transmission electron microscopy (TEM) analysis. The nanoemulgel was obtained by a gelification process of the characterized nanoemulsion. To evaluate the effectiveness of our formulation, we conducted a detailed in vivo study using rat models. This research specifically focuses on comparing the wound healing effects of the nanoemulgel. Our observations aim to provide insights into the therapeutic potential of the nanoemulgel, highlighting its efficacy in promoting wound healing and its advantages over conventional treatments.

## 2. Results and Discussion

### 2.1. Chemical Composition of Opuntia ficus-indica Seed Oil

The chemical composition of the vegetable oil extracted from *Opuntia ficus-indica* (OFI) seeds is summarized in Table 1. This composition is characterized by a high fatty acid content. PPSO contains 17.10% saturated fatty acids (SFAs)—mainly palmitic acid C16:0 (13.69%) and stearic acid C18:0 (3.11%). Monounsaturated fatty acids (MUFA: 22.55%) are almost exclusively represented by oleic acid C18:1 (21.48%). Linoleic acid C18:2 (60.13%) dominates the fraction of polyunsaturated fatty acids (PUFAs: 60.33%), which make up the majority of the chemical composition of OFI seed oil. The ratio of fatty acids/unsaturated fatty acids ((FFA)/UFA) is 0.206, confirming a high level of UFA.

A comparative study of the chemical composition of prickly pear seed oils harvested in Tunisia, Algeria, Morocco, Egypt, Saudi Arabia, and Mexico (summarized in Table 2) showed that these vegetable oils have similar palmitic acid concentrations (11.02–13.69%), except for that grown in Saudi Arabia, the concentration of which is 6.73%. The percentage of stearic acid varies between 3.11% and 5.74%. Oleic acid ranges from 19.99% to 22.41%, except in Morocco, where it represents only 6.73% of all identified monounsaturated fatty acids. Linoleic acid is the most abundant compound in the chemical composition of the oil extracted from prickly pear seeds harvested in Tunisia, Algeria, Morocco, and Egypt, with a content ranging from 54.04% to 66.79%, but its level is 14.00% in the fraction obtained from OFI growing in Saudi Arabia, which is dominated by linolenic acid (50.69%). These differences in the fatty acid contents of OFI oil could be linked to several factors, such as the month of harvest, climate [33], geography of the terrain [34], degree of maturity of the plant species [34], plant species management, and genetic factors that intervene directly or indirectly.

### 2.2. Evaluation of Prepared Nanoemulsions

The formulations were prepared with a 10% oil mixture (PPSO: IPM; 1:9) and a 15% surfactant mixture composed of Tween 80 and Span 80 with different HLB values. The HLB values of a surfactant mixture have an important role in emulsion formulation. During the generation of an oil-in-water emulsion, the lipophilic surfactant exhibited a higher affinity toward the emulsified droplets within the emulsion than the hydrophilic surfactant. Maintaining an appropriate HLB value is crucial to preserve the balance between the hydrophilic and lipophilic components [38].

The emulsions associated with HLB values of 9 and 9.5 exhibited instability, evident in macroscopic alterations like creaming and phase separation, leading to their exclusion from further evaluation. Assessment of their particle size and PDI was unfeasible. Notably, the emulsions showed an opaque (white) appearance with HLB values of 10 and 10.5, while those with HLB values of 11, 11.5, and 12 appeared transparent or translucent.

As referenced in [39], nanoemulsions are characterized by droplet sizes typically ranging between 20 and 200 nm. Their inherent size imparts kinetic stability, protecting them from sedimentation, creaming, flocculation, and coalescence.

Analysis of the droplet sizes after 1 day of manipulation revealed that the formulations with HLB values of 10 (304.6 ± 12.58 nm) and 10.5 (254.39 ± 10.33 nm) were categorized as macroemulsions. Meanwhile, the formulations with HLB values of 11 (56.46 ± 1.12 nm), 11.5 (101.04 ± 4.72 nm), and 12 (124.72 ± 8.56 nm) demonstrated characteristics typical of nanoemulsions.

**Figure 1:**
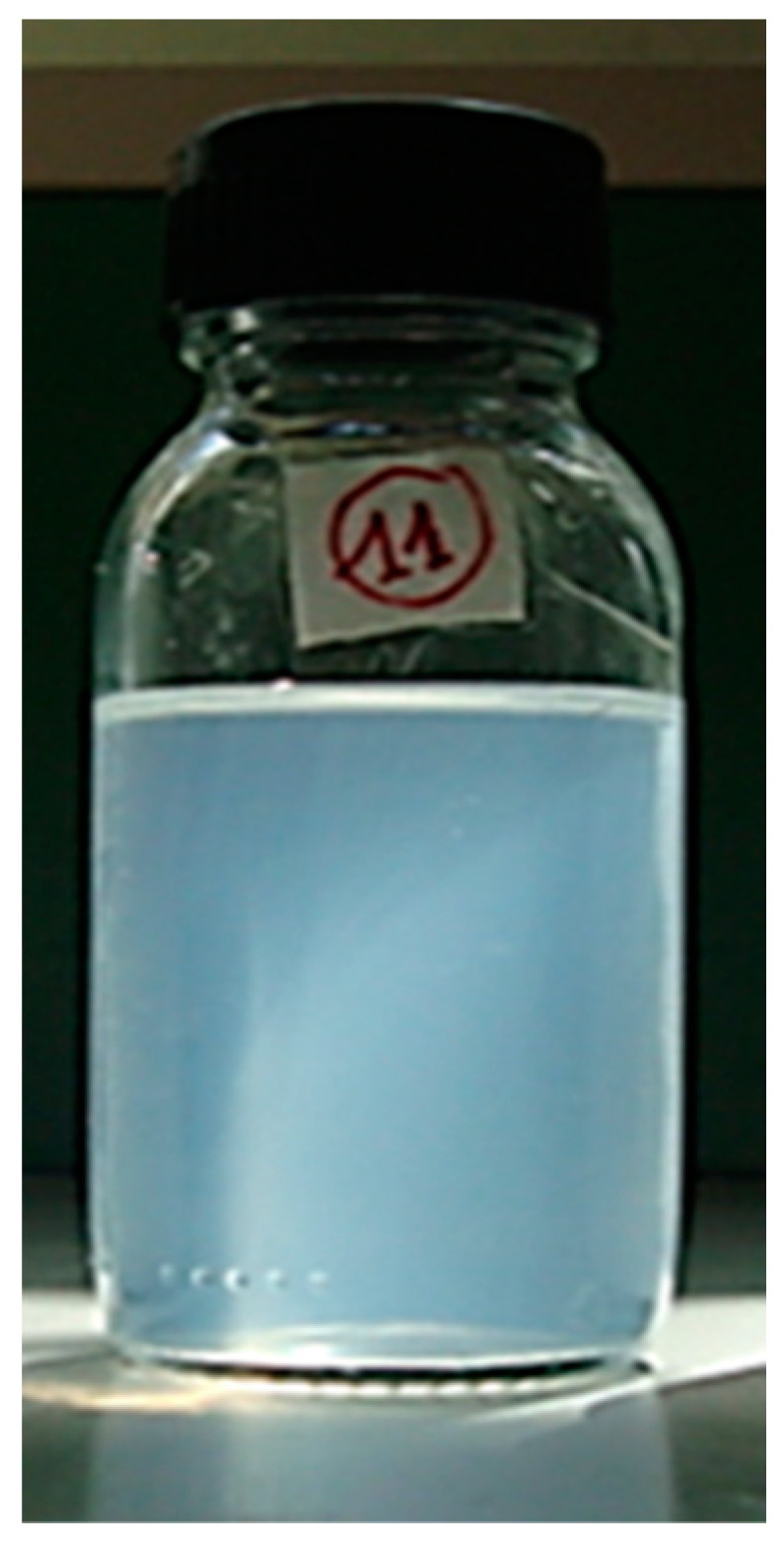
Optimal nanoemulsion (E5) corresponding to an HLB of 11.

The PDI of the emulsion formulations varied between 0.23 and 0.49. Notably, the lowest PDI (0.23 ± 0.01) was recorded at an HLB of 11, signifying excellent size distribution and an ideal monodispersed system for this nanoemulsion and coinciding with the smallest droplet size observed. Moreover, this formulation exhibited a bluish reflection, as shown in Figure 1, recognized as the Tyndall effect, a distinctive characteristic of nanoemulsions [40].

Based on the correlations among the minimum droplet size, the required HLB, and emulsion stability, it is suggested that an emulsion formulated with an HLB mixture closest to the required HLB of the oil mixture tends to exhibit the highest stability [39,41].

In this sense, we conducted a macroscopic evaluation of all formulations for up to 30 days. At the end of this storage period, the formulation possessing an HLB value of 11 exhibited no observable macroscopic alterations, retaining its initial fine appearance and bluish reflection. These findings imply that the optimal HLB value required for the utilized oil mixture may be close to 11. Consequently, we selected the corresponding formulation (E5) for subsequent experiments.

### 2.3. Characterization of the Selected Nanoemulsion

#### 2.3.1. Zeta Potential

The zeta potential serves as a crucial stability indicator for NEs and significantly influences their in vivo efficacy [42]. This potential reflects the charge difference between the surface of the droplets and the surrounding liquid medium, offering insights into the droplets’ surface charge within the medium [43]. It helps to reveal information about the repulsive forces among the droplets. Studies have indicated that for stable NEs, the zeta potential should exceed an absolute value of 25 mV [43]. Higher zeta potential values correlate with stronger repulsive forces between particles, mitigating tendencies toward flocculation or coalescence. When electrostatic and steric stabilization are used together, a minimum zeta potential of ±20 mV is considered advantageous [44]. Our preparation yielded a zeta potential of −31.4 mV ± 1.4 mV, suggesting exceptional stability in our optimized formulation.

#### 2.3.2. Transmission Electron Microscopy Results

**Figure 2:**
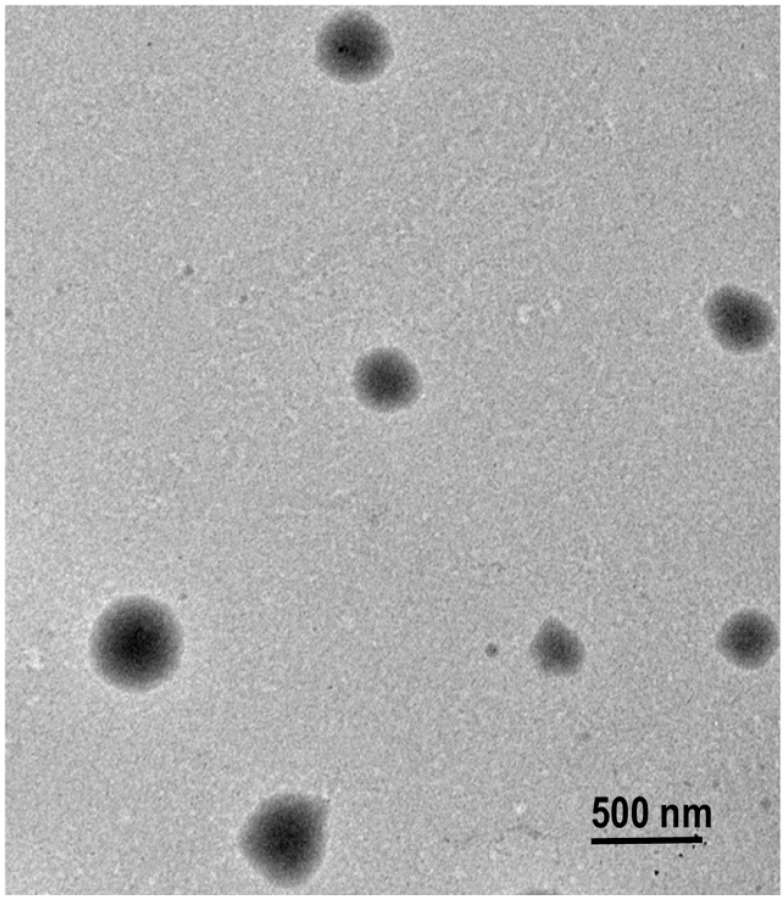
Surface morphology of the optimal NE using TEM.

The surface morphology of the selected nanoemulsion was analyzed using transmission electron microscopy (TEM). The images obtained show well-defined spherical droplets with almost no aggregation (Figure 2). These observations indicate sufficient homogeneity and stabilization of the droplets. These results, combined with the zeta potential data, suggest that the nanoemulsion prepared is stable within the nano-size range.

### 2.4. Nanoemulgel Preparation and Characterization

**Figure 3:**
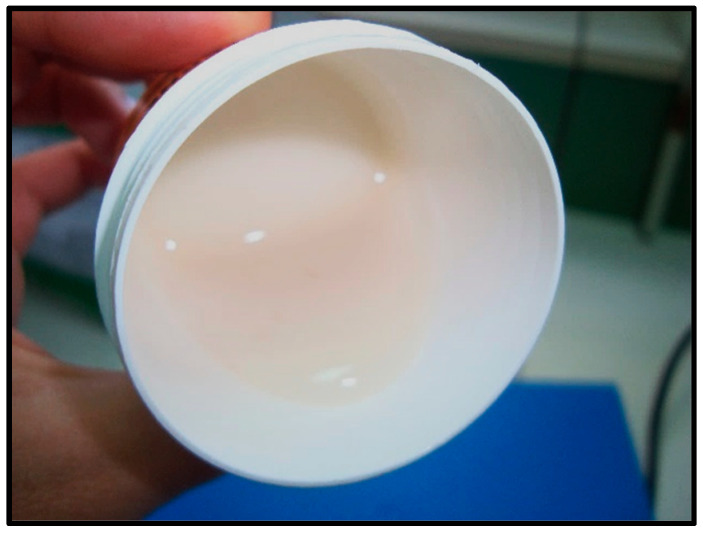
Visual aspect of the nanoemulgel loaded with PPSO.

The gelification process plays an important role in topical treatments using the selected prepared nanoemulsion. Due to its low viscosity, the initial liquid NE is not suitable for wound healing treatment, leading to formulation loss and reduced efficacy upon application. However, through gelification, the NE’s viscosity increases, which improves its adherence and suitability for efficient wound healing treatment [45]. Sepimax Zen^®^ was employed as the gel-forming agent, with a 1% concentration. The resultant nanoemulgel, depicted in Figure 3, revealed a uniform touch and a non-sticky feel. To optimize gelling, the choice of gel-forming agent proved crucial. Sepimax Zen^®^, a non-ionic polymer renowned for its thickening, stabilizing, and texturizing properties, facilitated the formulation of gels with a smooth, refined, and translucent texture.

#### 2.4.1. Measurement of the Nanoemulgel pH

The measured pH of 5.5 ± 0.1 falls within a satisfactory range and is unlikely to irritate the skin upon application. Human skin typically maintains an acidic pH between 4 and 6, crucial for preserving its integrity and enabling various essential processes, including keratinocyte differentiation and the formation of epidermal lipids [46]. Maintaining skin acidity is vital for homoeostasis and supports effective wound healing, as lower pH levels promote more efficient healing, while higher, alkaline environments are often associated with chronic wounds [47]. Additionally, an acidic pH fosters antimicrobial activity, which is crucial for preventing infections. Therefore, ensuring the formulation’s pH remains acidic, akin to the skin’s pH, is vital to safeguard the skin, maintain homoeostasis, promote optimal healing, and prevent infections.

#### 2.4.2. Viscosity Study of the Nanoemulgel

The measured viscosity of the nanoemulgel was determined to be 16,540 ± 45.28 centipoises (CP). This result suggests a moderately high viscosity, a common characteristic of nanoemulgel formulations, reflecting substantial resistance to flow, which can be advantageous for topical applications [48]. This observation aligns with various published studies, such as the work conducted by Alyoussef et al. (2021), who developed a nanoemulgel specifically for burn-induced wounds, demonstrating a similar viscosity range [2].

### 2.5. In Vivo Wound Healing Assay

**Figure 4:**
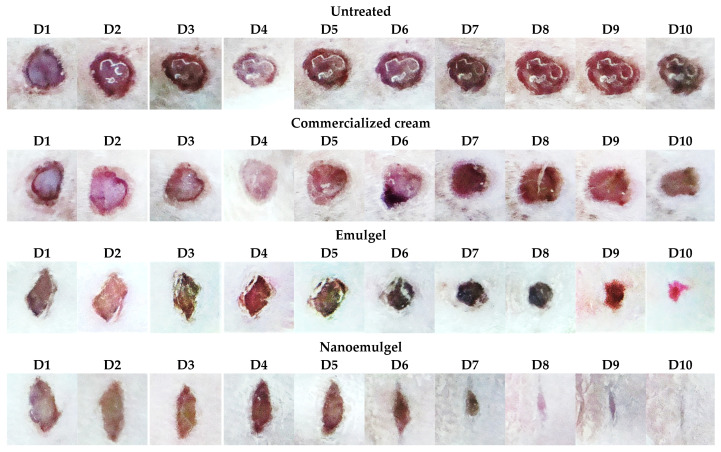
Daily wound images of the four groups (nanoemulgel, emulgel, commercialized cream cream, and untreated) over a 10-Day period (D1 to D10).

Wound contraction activity was studied to check the efficiency of the nanoemulgel formulated with PPSO compared to a conventional emulsion and a marketed cream. Wounds of all groups were monitored visually and digitally photographed each day, as presented in Figure 4. The wound areas were measured, and the percentage of wound closure was calculated, as mentioned before.

Regarding the nanoemulgel group, differences in the healing rates and wound closure compared to other groups were clear throughout the treatment. Three days after the injury, observations revealed a hard thrombus characterized by the presence of exudates over the area of injury in the rats in group 3 (non-treated). In contrast, among the other groups, group 1 (nanoemulgel) showed a relatively softer thrombus, with a marked reduction in inflammation and no discharge, followed by group 2 (conventional emulsion) and group 4 (marketed cream). Serious infection was not observed in any animal.

**Figure 5:**
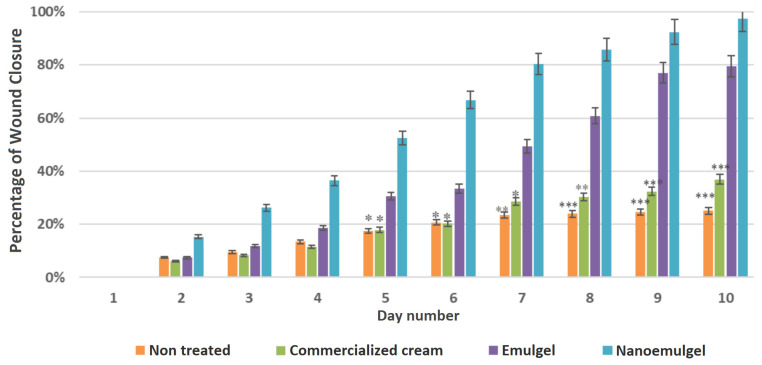
Daily percentages of wound contraction over the 10-day treatment period. Statistical significance was determined in comparison to the nanoemulgel group at each time point. Asterisks indicate levels of statistical significance: *p* < 0.05 (*), *p* < 0.01 (**), and *p* < 0.001 (***).

On day 5, the wounds treated with the nanoemulgel showed significantly higher wound closure, of 52.51%, compared to the conventional emulsion group and the one treated with the marketed cream (30.53% and 17.88%, respectively; *p* < 0.05), as represented in Figure 5 and summarized in Table 3.

The observation of granulation tissue formation occurred relatively early in the animals in the nanoemulgel group, as early as day 7. Remarkably, the healing process demonstrated a 1.6-fold acceleration compared to the conventional emulsion and a 2.8-fold acceleration compared to the marketed cream, highlighting significantly faster wound surface closure in the nanoemulgel group.

After 10 days, the nanoemulgel group wound area was significantly decreased with complete wound closure, whereas 79.39% healing was seen in the conventional emulsion group and 36.88% in the group treated with the marketed cream. Delayed wound healing was observed in the negative control group (25.10%).

In summary, the nanoemulgel formulation incorporating PPSO showcased notable effectiveness in promoting wound healing over 10 days. Our findings align with those of Khémiri et al., who conducted a study on the efficacy of free PPSO in wound healing, demonstrating its effectiveness by accelerating wound contraction, promoting complete re-epithelialization, and improving the external appearance of the scar. The outcomes were elucidated by active components of PPSO, such as unsaturated fatty acids, triacylglycerols, phytosterols, and tocopherols, which can exert their pharmacological effects individually or synergistically to enhance their efficacy [49].

In our case, the gel-based nanoemulsion demonstrated its superior efficacy to the gelified emulsion in terms of the wound healing rate, achieving complete re-epithelialization in a shorter period of 10 days. Indeed, the nanosizing process employed to incorporate bioactives into nanostructures would enhance their efficacy, safety, and stability. Additionally, the formulation’s higher viscosity, compared to low-viscosity oils, would ensure a comfortable application, preventing potential product loss.

Due to their nanometric size, the nanodroplets exhibit a significantly high surface-to-volume ratio, which may lead to modifications in physicochemical properties and potentially affect the pharmacokinetics and pharmacodynamics of the active principles [50]. This attribute could contribute to the observed enhancement in wound healing rates with the nanoemulsion.

This attribute would likely contribute to the superiority of the nanoemulsion in increasing wound healing rates. Furthermore, the typical Brownian movement of nanoemulsions would facilitate diffusion across the skin due to their kinetic activity, facilitating enhanced penetration into the deeper layers of the skin [51]. Lastly, the negative charge of nanodroplets (−31.4 mV) could provide better local concentration and restrict systemic leakage, which is undesirable in wound healing. For this purpose, the zeta potential would have an important impact on dictating localized effectiveness for wound healing [52]. 

Therefore, including PPSO in an oil-in-water nanoemulsion represents a superior alternative, especially when incorporated into an aqueous hydrogel system like nanoemulgels. This system offers advantages, such as even distribution, maintaining a moist environment, preventing wound dehydration, and facilitating oxygen penetration. These benefits create an optimal environment for effective wound healing [53]. However, while these results are promising, further studies are needed to validate the superiority of the nanoemulgel and to explore the underlying mechanisms in more detail. Several studies have demonstrated the benefits of using nanoemulgel systems in enhancing wound healing. For instance, Bahloul et al. (2023) reported similar improvements in wound closure rates using nanoemulsions containing essential oils [54], while Algahtani et al. (2021) observed enhanced bioavailability of the encapsulated drug and its therapeutic effects in topical applications of nanoemulgels [52]. 

Our findings are consistent with these studies, further confirming the potential of nanoemulgel formulations in wound care. However, our study uniquely highlights the efficacy of PPSO within this delivery system, thereby contributing novel insights to the field. However, it is important to note that this study was conducted using a single animal model, which may limit the generalizability of the findings to other species or human clinical settings. Future studies should consider multiple animal models or clinical trials to validate these results and ensure broader applicability.

In conclusion, the expedited healing process might be attributed to the synergistic effect of utilizing formulation nanotechnology to potentiate the active compounds within PPSO. Nano drug delivery systems, particularly nanoemulgels, have demonstrated their capability to enhance stability, bioavailability, and delivery mechanisms, significantly improving observed therapeutic efficacy [55].

While this study suggests a promising role for nanoemulgels in wound healing, the precise mechanisms underlying their enhanced efficacy remain to be fully elucidated. Potential mechanisms may include the improved penetration of active compounds due to the nanoscale size of the droplets, sustained release profiles, and enhanced interaction with the skin’s biological membranes. Future research should focus on uncovering these mechanisms in greater detail to better understand how nanoemulgels can be optimized for therapeutic use.

## 3. Conclusions

This study represents a major advancement in the treatment of wound healing with a nanoemulgel formulated with Tunisian prickly pear seed oil (PPSO). Adopting the high mechanical energy method and determining the required HLB, we developed a PPSO-based nanoemulsion, followed by gelation and in-depth characterization of its properties. The applied evaluation techniques, including TEM, zeta potential, pH analysis, and a viscosity assessment, provided information on the stability and characteristics of the nanoemulgel. This pioneering work has explored, for the first time, the use of a PPSO-based nanoemulgel in wound healing applications, opening up new perspectives in this area of research. Our in vivo study has confirmed the therapeutic efficacy of the developed formulation we have developed for wound treatment. Notably, its activity was observed in just 10 days, and this demonstrates its superiority over marketed wound healing creams. This innovative formulation shows great potential as a localized treatment. It underlines the importance of personalized topical drug delivery strategies, especially for wound healing. This research lays a solid foundation for future studies, including clinical trials to validate and apply these findings in practice. Our observations confirm previous findings on the healing properties of PPSO. This reinforces its potential as an efficient treatment. As the first study to explore nanoemulgels in this context, this work paves the way for important advancements in wound therapy.

## 4. Materials and Methods

Tween^®^ 80 (HLB = 15) and Span^®^ 80 (HLB = 4.3) were provided by Sigma-Aldrich (Tunis, Tunisia), Isopropyl myristate was obtained from Fluka Chemie (Buchs, Switzerland), and SEPIMAX ZEN^®^ was kindly provided by SMPC (Tunis, Tunisia).

The plant extract was prickly pear seed oil (PPSO), which was supplied by Herbalya^®^ (Monastir, Tunisia). The PPSO studied was an extra-virgin oil produced by cold pressing. It was solvent-free, dye-free, and preservative-free. Furthermore, it was certified by Ecocert^®^ (L’Isle Jourdain, France), certified with the organic farming label. It was a fluid at room temperature (15–30 °C) and relatively odorless, with a color ranging from light yellow to pale yellow. The oil was stored at +4 °C for later use.

### 4.1. Quantification of Fatty Acid in PPSO by Gas Chromatography

The fatty acid content was quantified by gas chromatographic (GC) analysis of the fatty acid methyl esters (FAMEs) in accordance with the AFNOR standards T60-233 and T60-234 [56]. 

#### 4.1.1. Preparation of Methyl Esters

The PPSO was treated with a 2 N methanolic KOH solution (potassium hydroxide). For this step, 2 mL of hexane and 3 mL of 2 N methanolic KOH were added to 1 g of oil. After being stirred for 30 s, the mixture was kept for 24 h until the upper phase became clear. This upper phase, containing the fatty acid methyl esters, was ready for analysis after dilution [57].

#### 4.1.2. Determination of Fatty Acid Methyl Esters by GC

The fatty acid methyl esters were separated and assayed by gas chromatography (GC). The chromatograph used was a Varian CP 3380 (Varian Inc., Palo Alto, CA, USA) equipped with a flame ionization detector and a capillary column packed with CPWAX 52 CB (Agilent Technologies, Santa Clara, CA, USA) as a stationary phase (length: 25 m; internal diameter: 0.25 mm; external diameter: 0.39 mm). The carrier gas was nitrogen; each sample was analyzed in triplicate. The fatty acid esters were quantified by comparing the retention times with those of the standards. These were the methyl esters of the subsequent acids: palmitic, heptadecanoic, stearic, arachidic, palmitoleic, heptadecenoic, oleic, gadoleic, linoleic, and linolenic [57].

### 4.2. Nanoemulsion Formulation

The PPSO-based NE was prepared using a high mechanical energy process, which is described in our recent research works [54,58].

The preformulation study and the team’s background were used to select the excipients and set the percentages of PPSO, oily vehicle, surfactant mixture, and water in the formulation.

All prepared NEs contained 1% PPSO (active oil), 9% IPM (oily vehicle), and 75% water. The emulsifiers Tween 80 and Span 80 were used in a total blend of 15% *w*/*w*. 

To determine the required HLB of the oily phase, the emulsions were prepared within a range of HLB (9 up to 12). Tween 80 (HLB = 14.9) and Span 80 (HLB = 4.3) were used in various ratios according to Park and Kim (2021) [26].

The percentages of co-surfactants and surfactants were calculated using the following equation [59].

%Tween 80 = [(X − HLB_Span80_) × 100]/(HLB_Tween80_  − HLB_Span80_) %Span 80 = 100 − %Span 80(1)

where X = the mixture’s HLB values of 9, 9.5, 10, 10.5, 11, 11.5, and 12.

Seven formulations were prepared (E1 to E7), and their compositions are shown in Table 4.

In summary, the process involved preparing two distinct phases heated separately to 50 °C. The first phase comprised PPSO, IPM, and the hydrophobic cosurfactant (Span 80). The second phase encompassed the aqueous components, including the surfactant (Tween 80) and deionized water. The latter was added slowly and incrementally to the oily phase, and then the obtained mixture was placed under a high-speed homogenizer, POLYTRON (KINEMATICA, Lucerne, Switzerland) for 2 min at 13,000 rpm. This rotor-stator homogenizer was equipped with a “G-GAS TIGHT” (KINEMATIKA, Lucerne, Switzerland) aggregate that features integrated mechanical seals. This setup included a 20 mm-diameter dispersion tool with a 210 mm shaft, suitable for processing volumes between 100 mL and 2000 mL. The preparation was conducted at room temperature. After preparation, the formulations were stored at room temperature for 24 h before characterization.

### 4.3. Characterization of Nanoemulsions

#### 4.3.1. Macroscopical Analysis

The assessment of formulation quality was conducted at intervals: immediately, and then after 1, 15, and 30 days of preparation, employing macroscopic observations to evaluate the color, visual appearance, phase separation, percentage of creaming, and sedimentation occurrence [39]. All emulsions were conserved throughout this duration in glass test tubes with screw caps at room temperature (25 ± 2 °C).

#### 4.3.2. Droplet Size Analysis

The droplet mean size and polydispersity index (PDI) of the nanoemulsions were assessed at 25 °C using a dynamic light scattering technique via the ZetasizerNano-S laser nanoparticle size analyzer (Malvern Instruments, UK). Each test was conducted in triplicate. The NE was diluted with distilled water (1/100). Subsequently, the dispersion of the nanodroplet sizes was determined.

#### 4.3.3. Measurement of Zeta Potential

The zeta potential corresponding to the selected nanoemulsion was determined by the Malvern Zetasizer (Malvern Instruments Ltd., Malvern, Worcestershire, UK). The nanoemulsion was diluted 1:100 with distilled water, and the analysis was automated, conducted three times at 25 °C for accurate measurements. The test was performed in triplicate.

#### 4.3.4. Transmission Electron Microscopy

The morphology analysis of the nanoemulsion was conducted using a JEM-100S electron microscope (JEOL Europe, Croissy-sur-Seine, France). The nanoemulsion (100 µL) was diluted 1:10, and then 2 µL was deposited onto a Carbon Formvar copper grid. After air drying, imaging was performed in the magnification mode to elucidate the shape and size of the nanoemulsion droplets. This methodology allowed for a comprehensive examination of the nanoemulsion’s structural features, providing crucial insights into its particle morphology and distribution.

### 4.4. Gelification Process and Nanoemulgel Characterization

The purpose of gelifying the nanoemulsion was to improve its utility in contrast to standard nanoemulsions, primarily by elevating its viscosity [60]. For gelification, 1% Sepimax Zen^®^ was employed as gel-forming agent (INCI: polyacrylate cross polymer-6). The Sepimax Zen^®^ was first weighed using an OHAUS precision balance (OHAUS, Parsippany, NJ, USA) and then sieved through a 500 µm mesh sieve (AS500, Retsch, Germany) to ensure a uniform particle size. The nanoemulgel formulation was carried out at room temperature (25° ± 2 °C). Sepimax Zen^®^ was gradually introduced into the nanoemulsion in small increments while being stirred with an IKA magnetic stirrer (IKA, Staufen, Germany) set at 400 rpm. The gel formation was achieved through continuous gentle stirring.

#### 4.4.1. pH Determination

The pH measurement of the nanoemulgel was conducted in triplicate by a pH meter. It was crucial for the formulation to exhibit a pH that corresponds to the naturally acidic pH of the skin, promoting compatibility to enhance its effectiveness while also preventing the onset of bacterial infections on the skin’s surface.

#### 4.4.2. Viscosity Measurement

To assess the viscosity of the nanoemulgel, measurements were carried out without dilution using a Brookfield DV III Ultra RV rheometer (Brookfield Engineering Laboratories, Inc., Middleboro, MA, USA), at a temperature of 25 °C ± 0.5 °C.

### 4.5. In Vivo Testing of Healing Activity

Four groups (G1, G2, G3, and G4) belonging to male Wistar rats were utilized, as detailed in Table 5. These rats exhibited a mean weight of approximately 200 ± 20 g. The animals were housed in a controlled laboratory environment with a regulated 12 h light/dark cycle. The temperature was maintained at 25 °C (±2 °C), with the relative humidity set between 45% and 55%. The rats were provided with a conventional diet of water and specially formulated small grains for their dietary needs. The animal study protocol was approved by the Ethics Committee of the Higher Institute of Biotechnology of Monastir (protocol code: CER-SVS/ISBM 002/2022; date of approval: 4 February 2022). All procedures were carried out with strict adherence to ethical guidelines to ensure the humane treatment of animals and minimize distress. Anesthesia was performed using isoflurane inhalation (1.5% isoflurane at a rate of 0.8 L/min). The rats were then placed on a heating pad to maintain their temperature at 37 °C.

The dorsal regions of the anesthetized rats were initially shaved using an electric trimmer, followed by thorough cleansing and disinfection of the skin to prevent infection. Three circular incisions, each measuring 10 mm in diameter, were then carefully made on the rats’ backs using a sterilized chisel and stainless steel divider. Throughout the procedure, the rats’ welfare was monitored closely, and analgesia was administered as needed to manage pain and discomfort. Wound progression was regularly monitored, with consistent daily photographs taken for 10 days. Fresh compresses and bandages were applied to the wounds to maintain a clean environment and facilitate healing. The formulations were uniformly applied over the entire lesion zone with a thickness of approximately 2 mm. The group assignments followed the delineation specified in Table 5.

This study adhered to the principles outlined in the PREPARE guidelines (‘Planning Research and Experimental Procedures on Animals: Recommendations for Excellence’) and the 3 Rs principles (replacement, reduction, refinement) to ensure ethical conduct. The group assignments were made following the delineation specified in Table 5.

The wound regions were quantified utilizing photography and ImageJ version 1.53f software for image processing (Image-J, U.S. National Institutes of Health). Each wound was photographed alongside a ruler, enabling subsequent calibration for accurate area measurements.

### 4.6. Statistical Study

The statistical analysis was performed using SPSS software version 16 (SPSS Inc., Chicago, IL, USA). The data were analyzed using one-way ANOVA to determine significant differences among the groups. The assumptions of one-way ANOVA, including the homogeneity of variances and the normality of data distribution, were tested using Levene’s test and the Shapiro–Wilk test, respectively. One-way ANOVA was followed by Tukey’s post hoc multiple comparison test to identify specific differences among the groups. The significance level was set at 0.05 (*p* < 0.05), indicating that differences were considered statistically significant if the *p*-value was less than 0.05.

## Figures and Tables

**Table 1:** Fatty acid composition (%) of PPSO.

Fatty Acids	Formula	Peak Area (%)
PPSO		
Saturated fatty acids		
Myristic acid	C14:0	0.11
Palmitic acid	C16:0	13.69
Margaric acid	C17:0	0.04
Stearic acid	C18:0	3.11
Arachidic acid	C20:0	0.15
Monounsaturated fatty acids		
Palmitoleic acid	C16:1	0.90
Heptadecenoic	C17:1	0.07
Oleic acid	C18:1	21.48
Gadoleic acid	C20:1	0.10
Polyunsaturated fatty acids		
Linoleic acid	C18:2	60.13
Linolenic acid	C18:3	0.20
∑SFA		17.10
∑PUFA		22.55
∑PUFA		60.33

**Table 2:** Percentages (%) of fatty acids in *Opuntia ficus-indica* seed oil growing in different countries.

Country	Governorate	Palmitic Acid (C16:0)	Stearic Acid (C18:0)	Oleic Acid (C18: 1)	Linoleic Acid (C18:2)	Linolenic Acid (C18:3)	Reference
Tunisia	National office of oil	13.69%	03.11%	21.48%	60.13%	-	
Tunisia	Nabeul	11.88%	03.64%	20.55%	61.42%	-	[33]
Tunisia	Sousse	11.08%	03.42%	21.26%	57.04%	-	[35]
	Kasserine	11.53%	03.41%	21.95%	58.14%	-	
Algeria	Souk Ahras	11.66%	03.18%	20.36%	62.63%	-	[36]
		11.87%	03.15%	19.99%	63.12%	-	
		11.69%	03.19%	20.14%	61.93%	-	
Morocco	Argan Oil Company, Casablanca	12.70%	03.36%	15.16%	66.79%	-	[37]
Egypt	Cairo Giza	17.11%	03.49%	22.41%	54.03%	-	[12]
Saudi Arabia	Taif	06.73%	05.74%	21.10%	14.00%	50.69%	[37]
Mexico	Mexican association CoMeNTuna, Hidalgo	12.88%	03.38%	17.06%	65.40%	-	[34]

**Table 3:** Percentages of wound closure in Wistar rats treated with different formulations over a 10-day period.

Day	1	2	3	4	5	6	7	8	9	10
Untreated	0.00%	7.48%	9.46%	13.40%	17.39% *	20.72% *	23.87% **	24.55% ***	25.10% ***	25.10% ***
Commercialized Cream	0.00%	6.13%	8.29%	11.34%	17.88% *	20.19% *	28.61% *	30.32% **	32.36% ***	36.88% ***
Emulgel	0.00%	7.42%	11.83%	18.66%	30.53%	33.33%	49.32%	60.82%	76.98%	79.39%
Nanoemulgel	0.00%	15.26%	26.21%	36.42%	52.51%	66.81%	80.28%	85.68%	92.41%	97.44%

Statistical significance was determined in comparison to the nanoemulgel group at each time point. Asterisks indicate levels of statistical significance: *p* < 0.05 (*), *p* < 0.01 (**), and *p* < 0.001 (***).

**Table 4:** Nanoemulsion formulations with varied HLB mixtures and ingredient ratios.

Formulation	HLB Mixture	PPSO (%)	IPM (%)	Tween 80 (%)	Span 80 (%)	Tween 80: Span 80 Ratio	Water (%)
E1	9.0	1	9	6.65	8.35	0.44: 056	75
E2	9.5	1	9	7.36	7.64	0.49: 051	75
E3	10.0	1	9	8.07	6.93	054: 046	75
E4	10.5	1	9	8.77	6.23	058: 042	75
E5	11.0	1	9	9.48	5.52	063: 037	75
E6	11.5	1	9	10.19	4.81	068: 032	75
E7	12.0	1	9	10.90	4.10	073: 027	75

**Table 5:** Evaluated formulations and their respective groups.

Group	Treatment
G1	Nanoemulgel
G2	Conventional emulgel
G3	No treatment
G4	Commercialized medicinal cream (API: hyaluronic acid) ‘Connettivina ^®^’

## Data Availability

The raw data supporting the conclusions of this article will be made available by the authors upon request.
